# Editorial: New Insights Into Behavioral Pharmacology

**DOI:** 10.3389/fnbeh.2021.754714

**Published:** 2021-09-24

**Authors:** Hugo Leite-Almeida, Nuno Sousa

**Affiliations:** ^1^School of Medicine, Life and Health Sciences Research Institute (ICVS), University of Minho, Braga, Portugal; ^2^ICVS/3B's - PT Government Associate Laboratory, Braga/Guimarães, Portugal; ^3^The Clinical Academic Center of Braga (2CA-Braga), Braga, Portugal

**Keywords:** behavior, EBPS young scientist workshop, School of Medicine/University of Minho, phenotypical analysis, neuropharmacology

In August 2019 we hosted at the School of Medicine/Life and Health Sciences research Institute (ICVS), University of Minho, the workshop (WS) “From networks to behavior and back—a EBPS Young Scientist Workshop.” It preceded the 2019 European Behavioral Pharmacology Society (EBPS) Biennial Meeting (Braga, Portugal) and the two events were brilliantly bridged by Professor Alcino Silva talk “Molecular Systems Neuroscience of Memory Linking”—see a recently published perspective by the group (De Sousa et al., 2021). The main aim of WS was to provide an encompassing view of the tools used to study behavior including (but not restricted to) the classical paradigms—see for an overview (Cunha et al., 2020)—and a number of techniques to manipulate in real time the experimental setting as a function of animals' performance—(Lopes and Monteiro); see also below—as well as to record/manipulate brain activity. The success of the WS was at all levels evident which prompted us to launch the “New Insights into Behavioral Pharmacology” research topic aiming primarily to resonate the spirit of the workshop.

Articles published under the scope of the research topic indeed tackled an array of behaviors ranging from pain (Jarrin et al.), aversive memory (Guilherme and Gianlorenco), depression (Patricio et al.; Surowka et al.), impulsivity (Esteves et al.), and drug seeking/abuse (Gyawali et al.; Konig et al.) as well as a number of systems and (patho)physiological processes that have profound implications in behavior including in the former monoaminergic (Amalric et al.; Guilherme and Gianlorenco) and endocannabinoid (Lujan and Valverde) systems and in the latter neurogenesis (Lujan and Valverde; Patricio et al.), epilepsy (Dare et al.) and neurodegeneration (Amalric et al.). While most of these studies used rodent models, particularly mice, Dare et al., describe a drosophila high throughput model (*para*^*bss*^ mutant) to test anti-epileptic drugs (Dare et al.). In addition to the obvious screening value that authors took advantage of, the trans-species validation of physiological and disease mechanisms can offer potentially new insights and avenues of research.

In a different perspective, but of a wide interest for behavioral neuroscience, Lopes and Monteiro, introduce readers to the principles and applications of the visual programming language Bonsai (Lopes et al., 2015), an open access tool that permits the simultaneous control of different data streams. They provide the reader with a number of examples and step-by-step tutorials that can be readily implemented by researchers with elementary programming competences. Specifically, when applied to behavioral settings, Bonsai can be used to extract in real time, relevant information regarding animals' behavior (e.g., position, movement, interaction with elements of the setting). More importantly, it can be used to precisely pair electrophysiological information with behavioral readouts. On the top of that, the system can be programmed to trigger instructions as a function of behavior. In this regard possibilities are immense ranging from the presentation of a cue, delivery of a reward or even optogenetic activation just to name a few.

All in all “New Insights into Behavioral Pharmacology” provides an holistisc view of the present Behavioral Neuropharmacology field. It projects to the future new capabilities that will help researchers to navigate through the complexity of many of todays' neuroscience questions that some years ago seemed technically out of reach. Animals' behavior is a powerful tool in Neuroscience but claims of behavior to brain function causality are still often made on the basis of loose associations. In this vein, we hope that this research topic, challenged, even if to a small extent, a reductionist bias as it has been called by other authors (Krakauer et al., 2017)—see also (Yartsev, 2017). Finally, it was gratifying to receive and edit manuscripts from many laboratories across the globe, particularly from several WS attendees and faculty.

## Author Contributions

All authors listed have made a substantial, direct and intellectual contribution to the work, and approved it for publication.

## Funding

This work has been funded by National funds, through the Foundation for Science and Technology (FCT)—project UIDB/50026/2020 and UIDP/50026/2020.

## Conflict of Interest

The authors declare that the research was conducted in the absence of any commercial or financial relationships that could be construed as a potential conflict of interest.

## Publisher's Note

All claims expressed in this article are solely those of the authors and do not necessarily represent those of their affiliated organizations, or those of the publisher, the editors and the reviewers. Any product that may be evaluated in this article, or claim that may be made by its manufacturer, is not guaranteed or endorsed by the publisher.
